# Follicular helper T cells recruit eosinophils into host liver by producing CXCL12 during *Schistosoma japonicum* infection

**DOI:** 10.1111/jcmm.14950

**Published:** 2020-01-07

**Authors:** Xiaojun Chen, Zhipeng Xu, Chuan Wei, XiaoWei Yang, Lei Xu, Sha Zhou, Jifeng Zhu, Chuan Su

**Affiliations:** ^1^ Department of Pathogen Biology and Immunology State Key Lab of Reproductive Medicine Jiangsu Key Laboratory of Pathogen Biology Center for Global Health Nanjing Medical University Nanjing China

**Keywords:** CXCL12, follicular helper T cells, recruitment of eosinophils, schistosomiasis

## Abstract

Schistosomiasis affects at least 200 million people in tropical and subtropical areas. The major pathology of schistosomiasis is egg‐induced liver granuloma characterized by an eosinophil‐rich inflammatory infiltration around the eggs, which subsequently leads to hepatic fibrosis and circulatory impairment in host. However, the mechanisms how eosinophils are recruited into the liver, which are crucial for the better understanding of the mechanisms underlying granuloma formation and control of schistosomiasis, remain unclear. In this study, we showed that follicular helper T (Tfh) cells participate in recruitment of eosinophils into liver partially by producing CXCL12 during schistosome infection. Our findings uncovered a previously unappreciated role of Tfh cells in promotion of the development of liver granuloma in schistosomiasis, making Tfh‐CXCL12‐eosinophil axis a potential target for intervention of schistosomiasis.

## 1 INTRODUCTION

Schistosomiasis remains a major public health problem in many developing countries in tropical and subtropical areas, which affects approximately 200 million people worldwide.^1^, ^2^ The egg‐triggered granulomatous responses in the liver, which lead to hepatic fibrosis and circulatory impairment, are central to the morbidity and mortality in schistosome‐infected humans.^3^ Understanding the mechanism of granuloma formation is crucial for a better treatment of schistosomiasis.

Eosinophils are recruited specifically to the developing hepatic egg‐induced liver granuloma and form a prominent constituent of the granuloma.^4^, ^5^ Indeed, the dramatic eosinophil infiltration is a dominant feature of the schistosoma granuloma.^6^ More importantly, eosinophils have been shown to be an important in vivo source of IL‐4 to maintain the Th2 response to schistosome infection,^7^ which is central to the development of hepatic granuloma and fibrosis.^8^, ^9^ In addition, eosinophils also contribute to CD4^+^ T cell responses by producing chemokines such as CCL17, CCL22, CXCL9 and CXCL10, which orchestrates the schistosoma granuloma formation.^10^, ^11^ Thus, studying the mechanisms underlying the recruitment of eosinophils into the liver provides additional insights into granuloma formation and novel strategies for schistosomiasis treatment.

In our previous study, we showed that follicular helper T (Tfh) cells play an obvious central role in hepatic granuloma formation during *Schistosoma japonicum* (*S* *japonicum*) infection.^12^ However, the precise pathogenic mechanism by which Tfh cells promote the granuloma formation remains to be determined. Here, we further demonstrated that Tfh cells recruit eosinophils into liver partially through producing CXCL12 during *S japonicum* infection. Our findings uncovered a novel mechanism of Tfh cells in the recruitment of eosinophils in schistosomiasis, which contributes to a deeper understanding and may help to design better treatment of the schistosomiasis.

## 2 MATERIALS AND METHODS

### 2.1 Mice and infection

C57BL/6J mice and inducible costimulator ligand (ICOSL)^−/−^ C57BL/6J mice were obtained from the SLAC Laboratory (Shanghai, China) and the Jackson Laboratory (Bar Harbor, ME), respectively. Mice were kept under specific pathogen‐free conditions and were used at 8 weeks of age. In the infection experiments, mice were infected percutaneously with 12 *S* *japonicum* cercariae obtained from infected *Oncomelania hupensis* snails purchased from the Jiangsu Institute of Parasitic Diseases (Wuxi, China). Animal experiments were performed in accordance with the Regulations for the Administration of Affairs Concerning Experimental Animals (1988.11.1). All animal procedures were approved by the Institutional Animal Care and Use Committee (IACUC) of Nanjing Medical University for the use of laboratory animals (Permit Number: NJMU 09‐0163).

### 2.2 Flow cytometry

Single‐cell suspensions from spleen, mesenteric lymph nodes and liver were isolated in phosphate‐buffered saline (PBS) containing 1% foetal bovine serum as described previously.^12^ For CXCL12^+^ Tfh cell analysis, cells were stimulated for 6 hours in culture medium containing phorbol myristate acetate (PMA, 25 ng/mL; Sigma‐Aldrich), ionomycin (1 μg/mL; Sigma‐Aldrich) and monensin (Golgi Stop; 1 μg/mL; BD Biosciences). Cells then were incubated for 30 minutes at 4°C with the following monoclonal antibodies: CD3e‐Percp‐cy5.5 (clone 145‐2C11, eBioscience), CD4‐PE‐Cy7 (clone GK1.5, eBioscience), PD‐1‐PE (clone J42, eBioscience) and CXCR5‐FITC (clone 2G8, BD Pharmingen). After staining of surface markers, the cells were permeabilized with cold Fix/Perm Buffer, and incubated with CXCL12‐APC (clone 79018, R&D Systems) after blockade with antimouse CD16/32 (clone 93, eBioscience). For eosinophil surface marker analysis, cells were incubated with Siglec‐F‐PE (clone E50‐2440, BD Pharmingen), CXCR4‐FITC (clone 2B11/CXCR4, BD Pharmingen) or isotype (BD Pharmingen) antibodies. Cells acquisition was performed using a FACSVerse cytometer (Lasers: 488 and 633; Mirrors: 507 LP, 560 LP, 665 LP, 752 LP, 660/10, and 752 LP; Filters: 488/15, 527/32, 568/42, 700/54, 783/56, 660/10 and 783/56, BD Biosciences). Data were analysed with FlowJo (Tree Star, version 10.0.7).

### 2.3 Adoptive transfer experiment

Fresh total cells from spleens of WT mice 8 weeks after infection with *S* *japonicum* were pre‐sorted by CD4^+^ T cell negative‐isolation kit (Miltenyi Biotec), and then stained with CD3e‐Percp‐cy5.5 (clone 145‐2C11, eBioscience), CD4‐FITC (clone GK1.5, eBioscience), PD‐1‐PE (clone J42, eBioscience) and CXCR5‐APC (clone 2G8, BD Pharmingen) antibodies. CXCR5^high^PD‐1^high^ Tfh cells were FACS‐purified by using a FACSAria cell sorter (BD Biosciences) to investigate the effect of Tfh cells on the cellularity of granulomas in the liver. FACS‐sorted Tfh cells were resuspended in PBS and injected intraperitoneally (ip) into the ICOSL^−/−^ mice 5 weeks after *S* *japonicum* infection (3 × 10^6^ cells/mouse). A group of mice that did not receive T cells but PBS was used as an additional control (mock transfer). Mice were sacrificed 3 weeks after transfer.

### 2.4 Quantitation of cell populations in the granulomas

Livers were fixed in 10% neutral buffered formalin. Paraffin‐embedded sections were dewaxed and stained with haematoxylin and eosin. The relative number of eosinophils in the granulomas was calculated by microscopic analysis by randomly counting 200 cells (not including hepatocytes) in each granuloma. Ten sections for each mouse and five microscope fields for each section were counted.^13^

### 2.5 Transwell migration assay

FACS‐sorted Tfh cells or MACS‐sorted naïve CD4^+^ T cells by a Naive CD4^+^ T Cell Isolation Kit (Miltenyi Biotec) were placed in the bottom of tissue culture wells in the presence or absence of PMA (25 ng/mL; Sigma‐Aldrich) and ionomycin (1 μg/mL; Sigma‐Aldrich). Eosinophils from the liver were incubated with anti‐PE microbeads (Miltenyi Biotec) after staining with Siglec‐F‐PE (clone E50‐2440, BD Pharmingen) and purified by MACS. Then, transwell inserts with MACS‐purified eosinophils (>95% pure) were placed on top. All of the transwell experiments were repeated three times with 3 wells for each treatment. The percentage of migrated eosinophils in each group was determined using a FACSVerse cytometer (BD Biosciences).

### 2.6 RNA isolation and reverse transcriptase polymerase chain reaction

Total cellular RNA was extracted using TRIzol (Invitrogen) according to manufacturer's instructions and quantified using a Biophotometer (Eppendorf). Reverse transcriptase reaction was performed using RevertAid First Strand cDNA Synthesis Kit (Fermantas Life Sciences). Primers sequences were as follows: CXCR4‐Forward AGCCTGTGGATGGTGGTGTTTC, CXCR4‐Reverse CCTTGCTTGATGACTCCCAAAAG; GAPDH‐Forward ACCACAGTCCATGCCATCAC, GAPDH‐Reverse TCCACCACCCTGTTGCTGTA. PCR conditions were 5 minutes denaturation at 94°C, 32 cycles of 30 seconds at 94°C, 30 seconds at 55°C and 1 minute at 72°C and 7 minutes elongation at 72°C in a Thermal Cycler. Amplicons of 242 or 452 base pairs were analysed by electrophoresis in 1% agarose gel and visualized using UV fluorescence after staining with ethidium bromide. All reactions were conducted with a negative control to ensure no contamination.

### 2.7 Immunohistochemical analysis

Livers were harvested from infected or normal mice and fixed in 10% neutral buffered formalin. Paraffin‐embedded sections were incubated with anti‐mouse CXCL12 (Cat 14‐7992‐81, Thermo Fisher Scientific) or isotype antibodies (Thermo Fisher Scientific), followed by an HPR‐conjugated secondary antibody. DAB was used as the substrate. All images were captured using an Axiovert 200M microscope (Carl Zeiss).

### 2.8 Statistical analysis

Data were expressed as the mean ± standard deviation. Student's *t* test was applied for comparisons between two groups, and one‐way ANOVA followed by LSD *t* test was used to compare differences between more than two different groups (GraphPad Prism software, version 5.01). *P*‐values of less than .05 were considered as statistically significant.

## 3 RESULTS

### 3.1 Tfh cells promote hepatic granuloma formation and control liver eosinophil number in *S japonicum*‐infected mice

**Figure 1:**
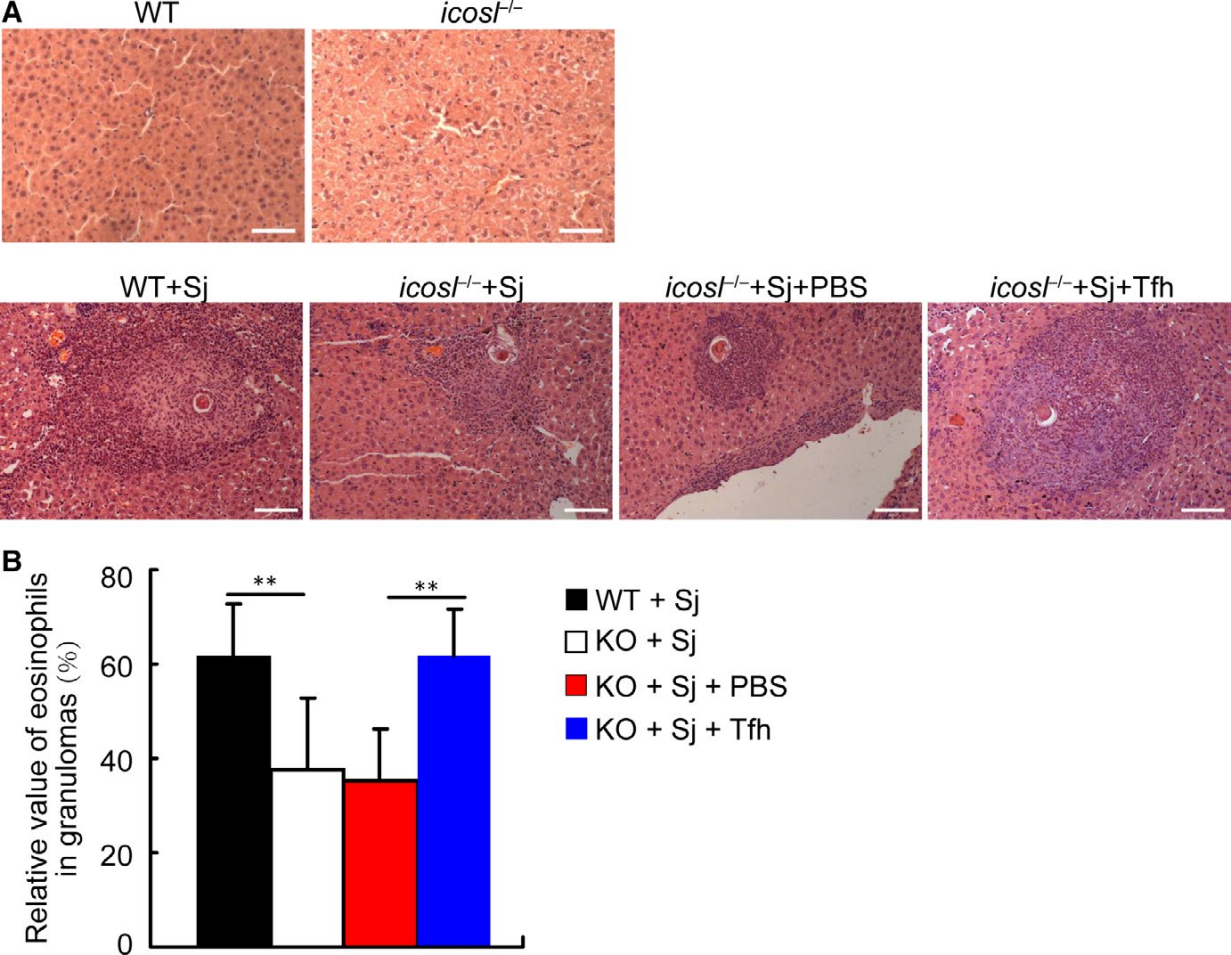
Tfh cells control granuloma formation and eosinophil number in the liver in *Schistosoma japonicum*‐infected mice. (A) Three weeks after adoptive transfer of Tfh cells and PBS, liver sections from *S japonicum*‐infected ICOSL KO recipient mice were stained with H&E. Images shown are representative of two independent experiments. Scale bars, 100 μm. (B) The relative number of eosinophils in the granulomas was determined by microscopic examination (1000× magnification) of 200 randomly selected cells (not including hepatocytes) in each granuloma. Values are given as mean ± SD of 12 mice from two independent experiments, **P < .01

Although our previously published data documents that Tfh cells represent a key pathogenic player in schistosomiasis japonica,^12^ little is known about how Tfh cells promote the granuloma formation. Given that the granuloma is characterized by the eosinophil‐rich granulomatous lesions,^4^ we wondered whether Tfh cells promote the granuloma formation through recruiting the eosinophils into the liver during schistosome infection. In ICOSL knockout (KO) mice, a widely used Tfh cell deficiency model,^12^, ^14^, ^15^ we showed a distinct reduction in the cellularity of granulomas surrounding single schistosome eggs after *S* *japonicum* infection (Figure 1A). Notably, the relative number of eosinophils in granulomas was substantially reduced in Tfh cell‐deficient mice (Figure 1B).

To further investigate the contribution of Tfh cells to the increase in eosinophils in the liver of *S japonicum*‐infected mice, Tfh cells were purified from *S japonicum*‐infected WT mice and adoptively transferred into Tfh cell‐deficient mice 5 weeks after infection. Compared with PBS group, the relative number of eosinophils in granulomas was significantly increased after adoptive transfer of Tfh cells into infected Tfh cell‐deficient mice (Figure 1B). These results indicate that Tfh cells contribute to the increase of eosinophil numbers in liver during schistosome infection.

### 3.2 Tfh cells recruit the eosinophils in vitro

**Figure 2:**
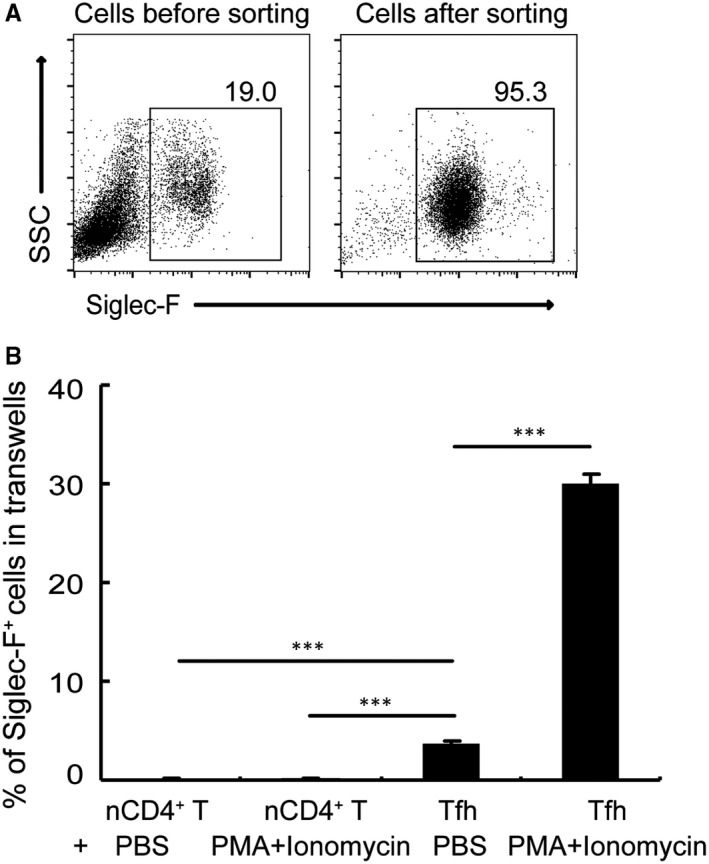
Tfh cells promote the migration of eosinophils in vitro. (A) The purity of MACS‐purified eosinophils was >95%. (B) In a transwell system, Tfh cells or naïve CD4^+^ T cells were cultured in triplicate wells in the lower chambers in the presence or absence of PMA and ionomycin. Eosinophils were isolated from the liver and were cultured in the upper chambers. Statistics show the percentage of Siglec‐F^+^ cells in total cells within the lower chamber. ***P < .001

To investigate whether Tfh cells have the potential to recruit eosinophils, we performed the in vitro transwell migration assay. Tfh cells were sorted with a FACSAria and placed in the bottom of tissue culture wells and transwell inserts with MACS‐purified eosinophils (>95% pure) were placed on top (Figure 2A). Compared with naïve CD4^+^ T cells, Tfh cells were able to stimulate eosinophil migration (Figure 2B). Treatment with PMA and ionomycin markedly enhanced the capacity of Tfh cells to recruit eosinophils in vitro (Figure 2B). Taken together, these results suggest that the soluble proteins secreted by Tfh cells may be involved in this recruitment process.

### 3.3 Eosinophils express CXCR4 in *S japonicum*‐infected mice

**Figure 3:**
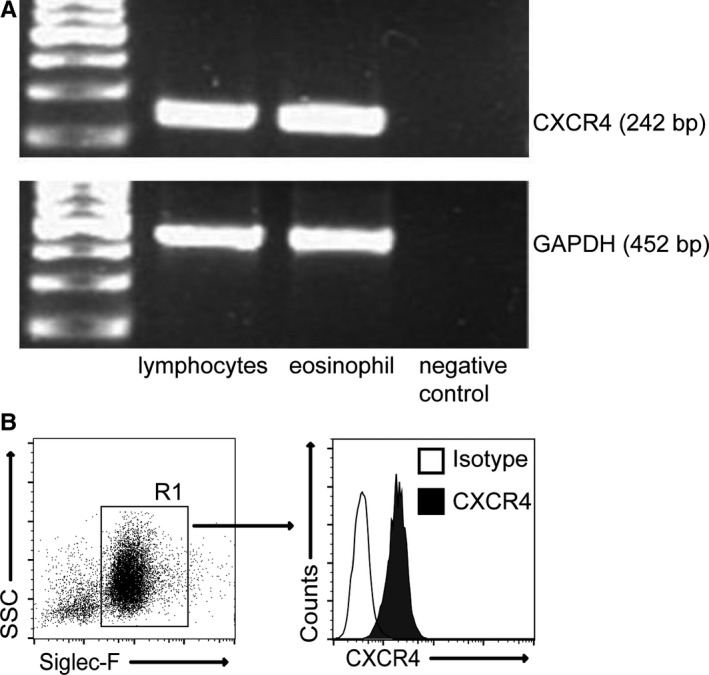
Eosinophils from schistosome‐infected mice express CXCR4. (A) Agarose gel electrophoresis of PCR products. Lane 1, Ladder DNA fragment marker; Lane 2, PCR products of CXCR4 and GAPDH from lymphocytes; Lane 3, PCR products of CXCR4 and GAPDH from eosinophils isolated from the liver; Lane 4, negative control (reaction without DNA). (B) Livers from infected mice were harvested, and cells were stained with Siglec‐F‐PE, CXCR4‐FITC or isotype antibodies. The level of CXCR4 on Siglec‐F^+^ cells was analysed

Given that CXCR4‐mediated signalling has important roles in both eosinophil recruitment in the context of allergic disease^16^ and granuloma formation in schistosomiasis,^17^ we performed RT‐PCR to test whether eosinophils in the liver of mice infected with *S japonicum* express CXCR4 transcripts. Results showed that CXCR4 transcripts were detected in eosinophil (lane 3), as well as in lymphocytes as a positive control because the lymphocytes have been reported to express CXCR4 in previous published paper^18^ (lane 2) (Figure 3A). More importantly, the surface expression of CXCR4 protein on eosinophils in the liver of infected mice was also clearly detected by flow cytometry (Figure 3B).

### 3.4 Tfh cells are an important in vivo source of CXCL12 in livers of mice with schistosomiasis japonica

**Figure 4:**
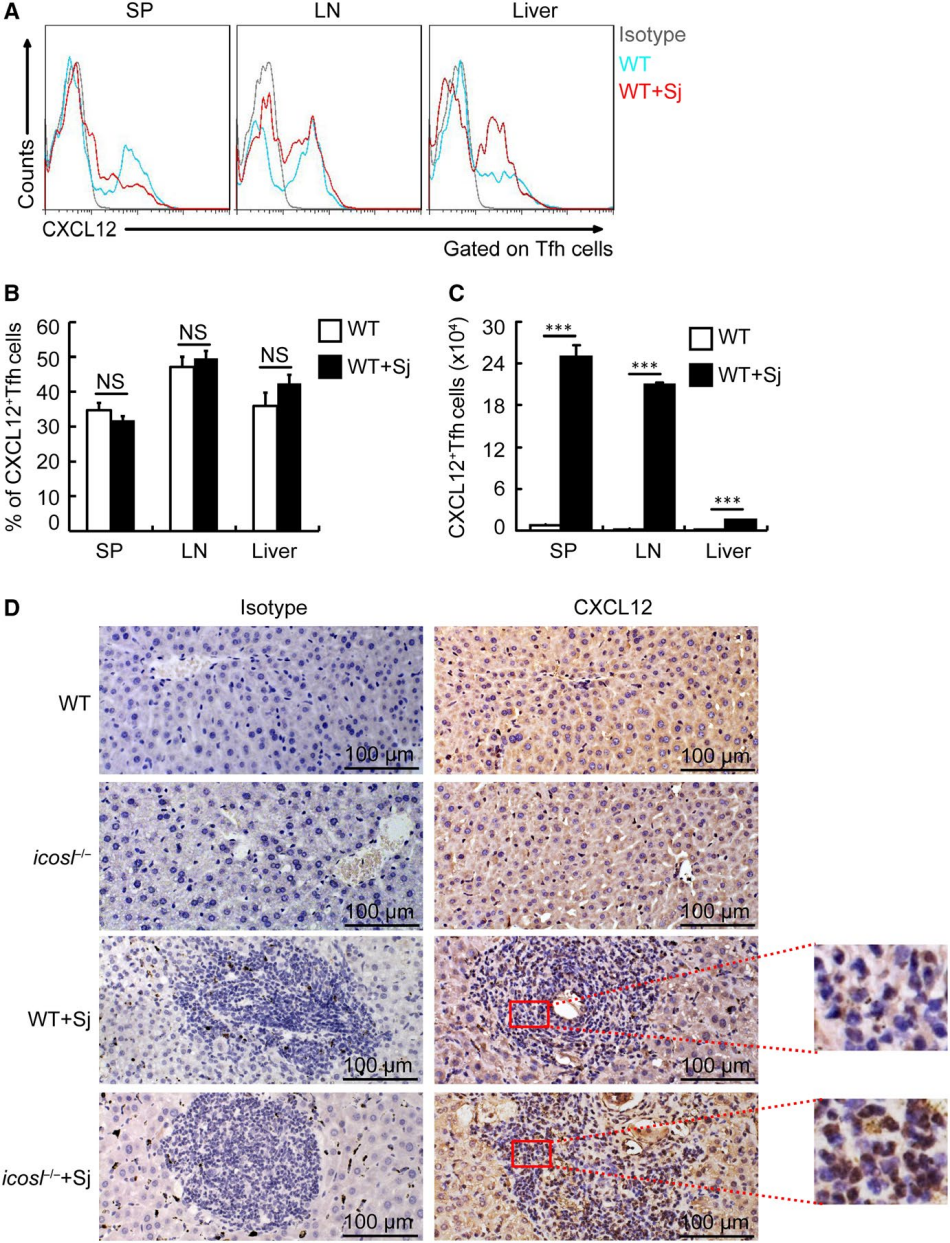
Tfh cells are an important source of CXCL12 in mice with schistosomiasis japonica. WT and *icosl* ^−/−^ mice were infected with 12 cercariae of *Schistosoma japonicum* per mouse, and sacrificed at 8 wk post‐infection. (A, B) Spleens, mesenteric LN and livers from normal and infected mice were harvested, and cells were stained with CD3‐percpcy5.5, CD4‐PE‐Cy7, CXCR5‐FITC, PD‐1‐PE and CXCL12‐APC antibodies. CXCL12^+^ cells were analysed and data shown are gated on CD3^+^CD4^+^CXCR5^+^PD‐1^+^ Tfh cells. (A) Representative flow cytometry data plots were shown; (B) statistics show the percentages of CXCL12^+^ Tfh cells in total Tfh cells. NS indicating not significant; (C) the absolute number of CXCL12^+^ Tfh cells was calculated. Data are expressed as the mean ± SD of 12 mice from three independent experiments, ***P < .001; (D) The liver tissue sections from WT or *icosl* ^−/−^ mice infected with or without *Schistosoma japonicum* were stained with anti‐CXCL12 or isotype antibodies. Scale bars, 100 μm

We next determine whether Tfh cells produced CXCL12, the principal ligand for CXCR4.^16^ Interestingly, 30%‐50% of Tfh cells constitutively expressed CXCL12 in spleen, lymph nodes and liver from normal mice or infected mice (Figure 4A,B). Although no difference in the percentages of CXCL12^+^ Tfh cells in total Tfh cells was observed in spleen, lymph nodes or liver between normal mice and infected mice (Figure 4B), the absolute number of CXCL12^+^ Tfh cells was markedly increased in mice after schistosome infection (Figure 4C). In addition, the expression of CXCL12 in the liver was considerably decreased in Tfh cell‐deficient–infected mice compared with WT‐infected mice (Figure 4D), suggesting Tfh cells as an important in vivo source of CXCL12 in liver in schistosome‐infected mice.

### 3.5 Tfh cells recruit the eosinophils partially dependent on CXCL12‐CXCR4 axis

**Figure 5:**
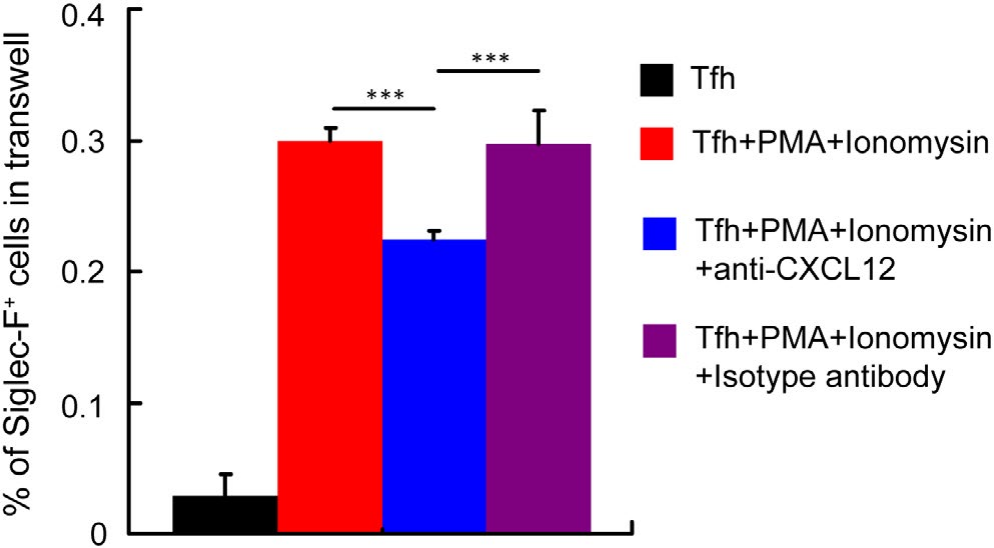
CXCL12 blockade reduce the ability of Tfh cells to recruit eosinophils. In a transwell migration system, Tfh cells were cultured in triplicate wells in the lower chambers in the presence of anti‐CXCL12 or isotype antibody with or without PMA and ionomycin. Eosinophils were cultured in the upper chambers. Statistics show the percentages of Siglec‐F^+^ cells in total cells within the lower chamber. ***P < .001

Finally, we investigated whether Tfh cells recruited eosinophils via producing CXCL12. We found that CXCL12 blockade by neutralizing antibody impaired the capacity of Tfh cells to recruit eosinophils in the in vitro transwell migration assay (Figure 5). Taken together, these results indicate that CXCL12 produced by Tfh cells contributes to the recruitment of eosinophils in the liver in *S japonicum*‐infected mice.

## 4 DISCUSSION

The eosinophil‐rich granulomatous lesions in the liver are the major contributors to the pathogenesis of schistosomiasis. Thus, clarification of the mechanisms responsible for the recruitment of eosinophils to the granuloma around schistosome eggs is important for understanding the formation of schistosome granulomas. In this study, we demonstrated that Tfh cells promote the migration of eosinophils and that chemokine CXCL12 produced by Tfh cells contributes to Tfh cell‐mediated recruitment of eosinophils during schistosome infection. Our findings provide additional insights into granuloma formation and potential therapeutic strategies for schistosomiasis.

Although eosinophils are not necessary for granuloma formation or liver fibrosis in mice following schistosome infection,^19^ they are not only a prominent granulomatous component but also an important source of IL‐4 to maintain the Th2 responses,^5^, ^7^ which supports eosinophils have a redundant but important role in granuloma formation in schistosomiasis.

Our previous paper has demonstrated that Tfh cells have a key role in the formation of hepatic granuloma in *S* *japonicum*‐infected mice.^12^ Although some studies raised the possibility that IL‐4 and/or IL‐21 produced by Tfh cells may contribute to Tfh cell‐mediated granuloma formation in schistosome‐infected mice,^20^, ^21^, ^22^ the exact mechanisms of how Tfh cells promote the granuloma formation remain unclear. The chemokine receptor CXCR4 and its chemokine CXCL12 are involved in the recruitment of immune and inflammatory cells.^23^, ^24^ Furthermore, evidence suggested that CXCR4 is expressed by circulating eosinophils from *Schistosoma mansoni*‐infected patients.^25^ It remains unclear, however, whether CXCL12 was involved in Tfh‐mediated eosinophil migration. In this study, we show that Tfh cells promote the migration of eosinophils in part through producing CXCL12, which may contribute to the granuloma formation. However, CXCL12 blockade in vitro only partially limits the ability of Tfh cells to recruit eosinophils, raising the possibility that the other chemokines, such as macrophage migration inhibitory factor (another ligand for CXCR4), eotaxins (ligands for CCR3), may be potentially involved in Tfh cell‐mediated migration of eosinophils.^26^, ^27^ In addition, the roles of IL‐5 in the proliferation, survival and migration of eosinophils have been investigated in the context of helminth infection.^28^, ^29^, ^30^, ^31^ Very recently, IL‐5‐producing Tfh cells have been identified in allergic disease,^32^ raising the possibility that one important mechanism of Tfh‐induced eosinophil migration may be through release of IL‐5. Indeed, these may explain why the blockade of CXCL12‐CXCR4 axis has only partially compromised Tfh cell‐mediated eosinophil recruitment in our study. Therefore, future studies will be crucial for identifying the other pathways of Tfh cell‐mediated eosinophil recruitment.

In summary, our study reveals a novel role of Tfh cells in controlling eosinophil migrating and provides additional insights into granuloma formation during *S japonicum* infection, making Tfh‐CXCL12‐eosinophil axis a potential target for treating schistosomiasis.

## CONFLICT OF INTEREST

The authors confirm that there are no conflicts of interest.

## AUTHORS' CONTRIBUTIONS

XC and CS conceived and designed the experiments. XC and CS analysed the data. XC, ZX, CW, XY, LX, SZ and JZ performed the experiments. CS and X.C wrote the paper. All authors read and approved the final manuscript.

## Data Availability

All data generated or analysed during this study are included in this published article.
